# Supplementary material for: Invasive species allelopathy decreases plant growth and soil microbial activity
Source: PLoS One. 2021 Feb 9;16(2):e0246685. doi: 10.1371/journal.pone.0246685 (PMC7872269; doi:10.1371/journal.pone.0246685)
Supplement: Attachment — Submitted filename: Response to Reviewers.docx [file pone.0246685.s001.docx]

**Response to Reviewers**

**Response to Reviewer 1**

**Question 1：**Author should explain how this root extract added to the soil can mimic what R. typhina is really releasing in the soil during its growth. In fact, the extract contains the molecules that water could extract from the whole root, but there is no proof that those molecules are the ones that *R. typhina* roots exudate in the soil. In the introduction, authors cite the “novel weapon hypothesis” and say that “Our results support the ‘novel weapons hypothesis’”. The cited hypothesis is proposed by Callaway and Ridenour (2004) who say “that some exotics transform from native weaklings to invasive bullies by exuding biochemicals that are highly inhibitory (allelopathic) to plants or soil microbes in invaded communities”. The hypothesis is based on molecules released as exudates. Also, the cited works in the discussion are about root exudates, rhizospheric soil, leaf leachates. Authors describe the effects on T. erecta growth and on soil bacteria, but those effect are due to molecules extracted from roots non exudated by roots. There is a big difference between root exudates and root extract.

**Answer：**Thank you for pointing out such a problem which we have indeed noticed that root exudates and root extract are really different as you said. Rhizosphere exudates are substances released by living roots during their growth, Broeckling et al. (2008) compared the effects root exudates of two model plant species (*Arabidopsis thaliana* and *Medicago truncatula*) on the soil fungal communities from various natural soils, which root exudate was obtained by vivo culture. Although plant species varied strongly in their root exudation pattern, plant root exudates were mainly water-soluble, for example root exudates were 79 % water-soluble in maize, including about 64 % carbohydrates, 22 % amino acids/amidesand 14 % organic acids (Birkettet al. 2001; Hütsch et al. 2002). Wu et al. (2001) obtained root exudates by aqueous solution extraction when they studied allelochemicals in wheat. As early as 1985, Gilmore (1985) studied allelopathy by means of aqueous extraction. Then This approach has been adopted by some researchers (Kil & Yun 1992; Ipo et al. 2012; Bao et al. 2012; Wang et al. 2018). From the above, we referenced these literatures to design experiments to mimic root exudates by root extract added to the soil although there are some drawbacks in this design.

Bao G. Saikkonen K, Wang H, Zhou L, Chen S, Li C, Nan Z (2015). Does endophyte symbiosis resist allelopathic effects of an invasive plant in degraded grassland? Fungal Ecology, 17, 114-125.

Birkett, M. A., K. Chamberlain,A. M. Hooper, and J. A. Pickett (2001): Does allelopathy offer real promise for practical weed management and for explaining rhizosphere interactions involving higher plants? Plant Soil 232, 31±39.

Broeckling CD, Broz AK, Bergelson J, et al. Root exudates regulate soil fungal community composition and diversity [J]. Applied and Environmental Microbiology, 2008, 74(3):738-744.

Hütsch, B.W., Augustin J., and Merbach, W. 2002. Plant rhizodeposition – an important source for carbon turnover in soils. J. Plant Nutr. Soil Sci. 165: 397-407.

Ipo, M., Blidar, C. F., & Bunta, D. (2012). Allelopathic effects of aqueous extracts from horseradish (*Armoracia rusticana* L.) metamorphosed roots on several cereals. Romanian agricultural research, 29(29).

Gilmore, A. R. . (1985). Allelopathic effects of giant foxtail on germination and radicle elongation of loblolly pine seed. Journal of Chemical Ecology, 11(5), 583-92.

Kil, B. S. , & Yun, K. W. . (1992). Allelopathic effects of water extracts of *Artemisia princeps* var. *orientalis* on selected plant species. Journal of Chemical Ecology, 18(1), 39-51.

Wang C,Wu B, Jiang K.(2018). Allelopathic effects of canada goldenrod leaf extracts on the seed germination and seedling growth of lettuce reinforced under salt stress. *Ecotoxicology* (London, England). Doi: 10.1007/s10646-018-2004-7

Wu, H, Haig, T, Pratley J, Lemerle D, An M. (2001). Allelochemicals in Wheat (*Triticum aestivum* L.): Production and Exudation of 2,4-Dihydroxy-7-Methoxy-1,4-Benzoxazin-3-One. Journal of chemical ecology. 27. 1691-700. Doi: 10.1023/A:1010422727899.

**Question 2：** Also, the choice of the control is not convincing. In my opinion, a more appropriate control would be not water but root extract from a plant that do not have an allelopathic effect on *T. erecta*, like one historically living in the same habitat. Authors should be sure that the effect on soil microbial activity is due specifically to *R. typhina* molecules and not to a general shift in microbial population due to the addition to the soil of any root extract.

**Answer：**Just as you say, we also considered using other non-allelopathic plants on T. erecta as controls when we designed the allelopathy experiment. However, we found that the choice of the control is water in previous studies on allelopathy (Broeckling et al. 2008; Ipo et al. 2012; Bao et al. 2012; Wang et al. 2018). Of course, we know that our experimental design cannot fully guarantee whether the effect is caused by allelochemicals or other components of the root extract, and we also know that it is an imperfect design in our experiment. We also consider that different plant extracts have different compositions which may interfere with experimental results in other ways, although there not allelochemicals in other plants (Birkettet al. 2001; Hütsch et al. 2002; Kato-Noguchi et al. 2014).

Kato-Noguchi, H., Moriyasu, M., Ohno, O., Suenaga, K. (2014). Growth limiting effects on various terrestrial plant species by an allelopathic substance, loliolide, from water hyacinth. Aquatic Botany, 117, 56-61.

**Question 3:** In some points of the discussion authors over-interpret their data:
**3.1:** lines 202-203: they state “Taken together, our results indicate how allelopathy impacts both plant and soil community, revealing that plant–soil microbe interactions can mediate invasion success”. I agree on the first part on the sentence, since root extract do have an effect on plant growth and on microbial population. However, data do not help us understanding whether the effect on plant is direct or mediated by microbes.

**Answer:** We agree with your advice and we revise this sentence to “Taken together, our results indicate how allelopathy impacts both plant and soil community further facilitates the successful invasion**”**.

**3.2:** Line 204: “our results show that plants have the potential to alter soil”: this sentence is misleading: written like this means that plants, in their active growth, can alter soil, but results are on root extract not on exudates.

**Answer:** We agree with your advice that we over-interpret our data, so this sentence “our results show that plants have the potential to alter soil properties, which may result in dramatic changes in soil community activity” is revised to “our results show that root extracts of *R. typhina* may result in dramatic changes in soil community activity by alter soil properties”.

**3.3:** Lines 206-207. “This study suggests that invasive species can increase their own fitness relative to other plant species by modifying soil conditions and microbial community” and Lines 238-239 “are the main factors shifting soil microbial activity and mediating the impact of invasive plants on the growth of other plants.” Again, there is not proof in the work that effect on plants is either direct or mediated by other factors.

**Answer:** We agree with your advice and delete the sentence (lines 206-207. “This study suggests that invasive species can increase their own fitness relative to other plant species by modifying soil conditions and microbial community”)；Then we also delete “and mediating the impact of invasive plants on the growth of other plants”.

**Question 4:** Citation of literature is sometimes not appropriate
**4.1:** Line 209: Tian et al 2017: this work is about the effect of N fertilisation on soil microbial community, not on allelopathy

**Answer**: Sorry about the wrong citation of literature and thank you point out this mistake, we delete this literature.

**4.2:** Lines 226-228: “These results are partially in agreement with previous studies indicating that root extracts serve as soil carbon substrates for specific soil microbial communities (Fan et al. 2010, Wu et al. 2013, Li et al. 2017)”. None of these cited papers deal about root extracts: they describe works on exudates and rhizospheric soil.

**Answer**: We realize the difference between root extracts and root exudates, so we also delete this sentence and their relevant literates.

**Question 5:** Minor points
**5.1:** Line 41: either remove “is”, or add “which” before “is”

**Answer**: “which” is added before “is”.

**5.2:** Line 64: Substitute “As” with “It is” or rephrase the sentence

**Answer**: “where it” is revised to “*R. typhina*”

**5.3:** Line 98: “five plant” modify in “five plants”

**Answer**: It has been revised.

**5.4:** Line 137: remove “in”

**Answer**: It has been revised.

**5.5:** Line 138: change “concentration extracts” to “extract concentrations”

**Answer**: It has been modified.

**5.6:** Lines 146-147: according to Table 1, plant height is not significantly different from control at 10 mg/mL.

**Answer**: Sorry, we made this mistake and “plant height” has been modified.

**5.7:** Line 164: table 2 shows soil properties data not AWCD.

**Answer**: Sorry, we made this mistake and “table 2” has been modified to “Fig 2”.

**5.8:** Line 230: Olchowik et al. 2012 demonstrated the presence of tannins in leaves: there is no indication of their presence in roots
**Answer**: Thank you for your advice, and we delete this sentence.

**Answer to the Reviewer 2**

**Question:** However more information on the conditions in which *T. erecta* has been grown during the experimental condition, such as light humidity, temperature, photoperiod. More information about the use Community-level physiological profiles (CLPP) should be provided for reader who are not familiar with such method.

**Answer**: According to your advices, we add the experimental condition in Line 97-98 “a climate-controlled incubator at 25 °C with diurnal lighting (light intensity was set at 2200 Lux on a 14:10 h light–dark cycle)”.

Community-level physiological profiles (CLPP) are also supplemented and perfected in Line 120-124 “Soil samples (10 g d.w.) were firstly shaken (5 min, 180 r·min^-1^) after dilution with 90 mL of distilled sterile water (pH 7) for 20 min at 20 °C. Then aliquot (150 μL) were inoculated into wells of 96 Biolog Eco-plates and incubated at 25 °C. The rate of utilization was showed by the reduction of the tetrazolium, a redox indicator dye that changes from colorless into purple. Lastly, substrate utilization was monitored by measuring light absorbance at 590 and 750 nm”.
